# Regular consumption of green tea improves pulse pressure and induces regression of left ventricular hypertrophy in hypertensive patients

**DOI:** 10.14814/phy2.14030

**Published:** 2019-03-25

**Authors:** Ahmad I. M. Al‐Shafei, Ola A. A. El‐Gendy

**Affiliations:** ^1^ Istanbul Medipol University Istanbul Turkey; ^2^ Bahrain University Southern Governorate Bahrain; ^3^ Qassim University Qassim Saudi Arabia; ^4^ Ain Shams University Cairo Egypt

**Keywords:** Green tea, hypertension, left ventricular hypertrophy, pulse pressure

## Abstract

This study characterized the effects of regular green tea (GT) and hot water (HW) ingestion on systolic blood pressure (SBP), diastolic blood pressure (DBP), pulse pressure (PP), and left ventricular hypertrophy (LVH) in two equal, sex‐ and age‐matched groups; Grp1 and Grp2 (*n* = 100 each; age 53 ± 4 years) of hypertensive patients. Grp1 had regular GT treatment, followed by HW ingestion, whereas Grp2 had HW ingestion followed by GT treatment for periods of 4 months each. Electrocardiographic (ECG) and echocardiographic assessments of LVH were made before and at the end of both periods. SBP was lowered significantly by 6.6%; DBP by 5.1%, and PP by 9.1% by the end of month 4 of GT treatment in Grp1. Upon GT cessation and HW ingestion, SBP, DBP, and PP returned to pretreatment levels over 4 months. In Grp2, SBP, DBP, and PP were reduced insignificantly by 1.5%, 1.0%, and 2.3% by the end of the 4th month of HW ingestion. Conversely, over 4 months of GT treatment, SBP, DBP, and PP were significantly lowered by 5.4%, 4.1%, and 7.7% from the baseline values, respectively. ECG and echocardiographic evidence of LVH was shown in 20% of Grp1 and 24% of Grp2 patients before intervention. This was significantly lowered to 8% and 10% in Grp1 and Grp2 by GT treatment. However, this increased to 16% following HW ingestion in Grp1. HW ingestion did mot induce regression of LVH in Grp2. Thus, regular GT ingestion has cardiovascular protective effects.

## Introduction

Systemic hypertension causes multiple clinical complications including cardiovascular disease (CVD), heart failure, nephropathy, and retinopathy (Mancia et al. 2016; Willey et al. 2016; Qin et al. 2017).

The pulsatile component of the blood pressure curve is referred to as the PP. It is largely determined by left ventricular ejection and compliance of large arteries (Radchenko et al. 2016). Hypertension is currently defined and managed based on levels of SBP and DBP, but not PP (Said et al. 2018). However, there has recently been an increasing interest in the PP as an independent predictor of the high incidence of CVD, congestive heart failure, and mortality in hypertensive patients (Glasser et al. 2014, 2015; Said et al. 2018). As a person ages, arterial stiffening causes SBP to increase and DBP to decrease (Said et al. 2018). Thus, for middle‐aged and elderly subjects, PP may be a more accurate predictor of cardiovascular death than either SBP or DBP alone (Protogerou et al. 2007; Said et al. 2018). Interestingly, there is emerging evidence that PP is a strong indicator of cardiovascular risk even among normotensive persons (Fang et al. 2000; Mazza et al. 2001).

Thickening of the muscle of the left ventricle (LVH) is the major cardiac complication associated with the progressive pressure overload in hypertension (Okin et al. 2017). Although adaptive in generating greater forces of contraction, the associated stiffness of the ventricle leads to diminished diastolic compliance and impaired diastolic filling (Masugata et al. 2011). Eventually left ventricular systolic function becomes impaired; potentially leading to left ventricular failure (LVF) if ventricular afterload is not corrected (Hou and Kang 2012; Nepper‐Christensen et al. 2017). Patients with LVH show increased prevalence of cardiac arrhythmias, increased incidence of sudden death even in the absence of epicardial coronary arterial disease, and increased infarct size with decreased myocardial salvage in patients with ST‐segment elevation myocardial infarction (Nepper‐Christensen et al. 2017). Antihypertensive therapy should therefore be targeted not only at lowering blood pressure, but also at inducing regression or of LVH (Hou and Kang 2012; Okin et al. 2017).

Tea is the most widely consumed beverage after water. However, there has been relatively little research on the effects of green tea (GT) ingestion on blood pressure. GT has been reported to exhibit a hypotensive effect in rats (Potenza et al. 2007; Ihm et al. 2012; Coşan et al. 2015; Turgut Coşan et al. 2015; Szulińska et al. 2017). Conversely, human clinical trials on the effects of GT on blood pressure have reported conflicting results (Hodgson et al. 1999; Yang et al. 2004; Yarmolinsky et al. 2015). Interestingly, GT has been shown to attenuate LVH in SHR rats, renal hypertension and angiotensin II‐induced cardiac hypertrophy in rats (Papparella et al. 2008; Jin et al. 2017), and rats subjected to remnant kidney surgery (Priyadarshi et al. 2003).

This study reports for the first time, the effects of regular consumption of GT on PP and LVH, in patients with hypertension, and complements previous clinical and animal studies (Priyadarshi et al. 2003).

## Subjects and Methods

### Volunteers

This study was a randomized controlled investigation carried out at Kafr El‐Shaikh General Hospital, Egypt, for 8 months. The study complied with the Declaration of Helsinki with investigations conducted with the understanding and written informed consent of each participant and approval of the Research and Ethics Committees of Kafr El‐Shaikh General Hospital, Egypt. The study included hypertensive patients who are used to come to Kafr El‐Shaikh General Hospital for their regular checkups, and included both males and females with an average age of 53 ± 4 years. Potential volunteers were first requested to complete a screening questionnaire. Individuals who were regular consumers of tea or coffee, current smokers or ex‐smokers who had stopped smoking within the past 6 months, or who had any history of a major illness including liver, renal, gastrointestinal, or thyroid disease were excluded from the study. Inclusion criteria were a SBP of 150–180 mmHg and/or a DBP of 95–120 mmHg, use of two or fewer antihypertensive drugs and an absence of secondary hypertension. Participants were then randomly selected from eligible volunteers to have a total of 200 patients. All patients had their SBP, DBP, PP, and heart rate (HR), measured before starting the study in order to have baseline data for these parameters. Additionally, all subjects had an ECG and an echocardiography at the beginning of the study. The 200 patients were then randomly divided into two groups (Grp1 and Grp2) of 100 patients each (*n* = 100). Both groups contained equal numbers of males and females, and so patients from each group were carefully matched for age and sex. Selected participants were finally screened prior to participation and the study design and requirements were thoroughly explained to them.

### Study design

The study lasted for a total of 8 months. Patients in Grp1 were instructed to drink four standard cups (250 mL each) of green tea (GT) per day, and patients in Grp2 were informed to drink similar amounts of hot water (HW) in the same manner for the first 4 months of the study. SBP, DBP, PP, and HR were measured in the morning at the end of each month of intervention. GT treatment was then stopped in Grp1 for 4 months, and patients were instructed to drink four standard cups (250 mL each) of HW per day for the remaining 4 months of the study. On the other hand, patients in Grp2 were told to stop drinking HW and begin regular ingestion of four standard cups (250 mL each) of GT daily for the remaining 4 months of the study. All patients had the above‐mentioned parameters rechecked by the end of each month of the second 4 months period. An ECG and an echocardiogram were also done for all patients at the end of both periods. During the study, subjects were instructed to cease intake of caffeine‐containing beverages, and not to make any changes to their usual food intake, physical activity and treatment prescribed by their doctors for hypertension.

### Tea preparation

Lipton decaffeinated green tea was obtained from the local market. This brand of green tea contains about 130 mg of flavonoids (catechin and epicatechin) per 8 fl oz (250 mL) serving. The method of green tea preparation was standardized as far as possible. Subjects were instructed to use a standard cup (8 fl oz cup), which holds approximately 250 mL of water to prepare the tea. Each time a cup of green tea prepared, 1 tea bag (2 g) was added into 250 mL of boiled water for 1 min with constant movement. The drink was then consumed without additives, including milk and sugar.

### Blood pressure measurement and heart rate determination

SBP and DBP were measured using an Omron M7 (HEM‐780‐E) electronic sphygmomanometer (Omron Healthcare Co., Limited, Kyoto, Japan). Subjects rested for approximately 5 min. SBP and DBP were then measured on the left arm on three occasions at 5‐min intervals. The means of the measurements were used in subsequent analyses. PP was calculating by subtracting DBP from the SBP of every individual. Blood pressure measurements were not disclosed to participants during the study.

HR was determined by measuring the pulse rate manually and the results were compared with the values given by the electronic sphygmomanometer. There were no differences between pulse rate values determined by both methods.

### Electrocardiographic (ECG) and echocardiographic detection of LVH

Electrocardiograms (ECGs) and echocardiography were obtained according to protocol at the beginning and the end of each GT and HW 4 months’ intervention period. ECGs were interpreted by observers blinded to other clinical data. The Sokolow‐Lyon voltage criteria (S_V1_ + R_V5/6_) higher than 38 mm were used to identify LVH (Bacharova and Estes 2009; Okin et al. 2017) Echocardiography was done by cardiologists who were blinded to clinical data and ECG findings. Echocardiographic diagnosis of LVH was made based on the left ventricular mass index (LVMI), which was calculated from measurements of interventricular septum thickness, posterior wall thickness of left ventricle, and end‐diastolic left ventricular diameter (Lovic et al. 2014).

### Statistics

Statistical analyses were performed using SPSS software (SPSS, Chicago, IL, U.S.A.). Results are presented as means ± standard deviations from the means (SD). One‐way analysis of variance (one‐way ANOVA) followed by Tukey's test were used to analyze changes in SBP, DBP, PP and HR; ^aaa^ *P* < 0.001, ^aa^ *P* < 0.01 and ^a^ *P* < 0.05 (comparing values by the end of each month of the 8‐month study period with the baseline value); ^bbb^ *P* < 0.001, ^bb^ *P* < 0.01 and ^b^ *P* < 0.05 (comparing values after the 1st, 2nd, 3rd, and 4th months of the second period of intervention with the level by the end of the 4th month of the first period of intervention). LVH four months after GT or HW treatment was compared with baseline LVH and with LVH four months after reversing the GT and HW inventions in the two study groups using Fisher's exact test.; *P < 0.05, **P < 0.01 (comparing LVH by the end of the first intervention period with baseline LVH); ^+^ *P* < 0.05, ^++^ *P* < 0.01 (comparing LVH by the end of the second intervention period with LVH by the end of the first intervention period); ^§^ *P* < 0.05, ^§§^ *P* < 0.01 (comparing LVH by the end of the second intervention period with baseline LVH)

## Results

All the 200 patients had their baseline SBP, DBP, PP, and HR measured before starting the study. They were then randomly divided into the two study groups; Grp1 and Grp2. The two study groups therefore had identical baseline values for the investigated parameters. Mean SBP, DBP, and PP values were 163.5 ± 8.1, 102.9 ± 8.7, and 60.6 ± 10.0 mm Hg, respectively. Baseline HR was 68 ± 7 beat/min.

**Figure 1:**
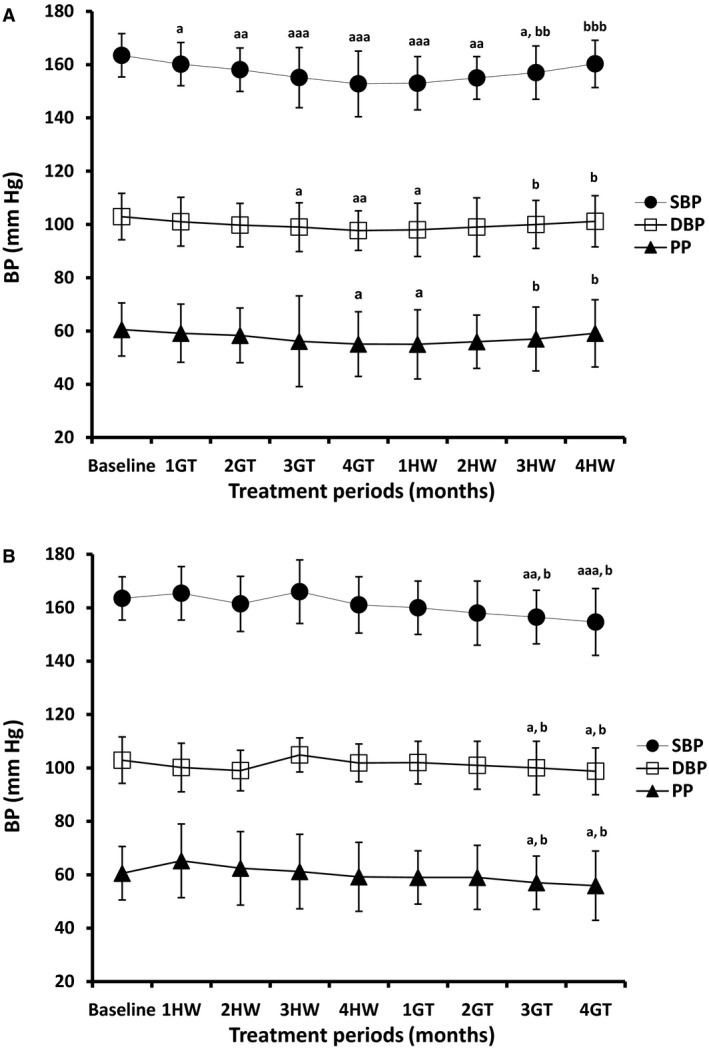
Effects of GT treatment and HW ingestion on SBP, DBP, and PP (mmHg). Changes in SBP, DBP, and PP for Grp1*(1A)* and Grp2 *(1B)* following two periods of GT and HW intervention for 4 months each; Grp1 had GT treatment followed by HW ingestion and Grp2 had HW ingestion followed by GT treatment. During each intervention period, patient were instructed to drink either four standard cups (250 mL each) of GT or HW per day for 4 months from left to right in sequence. 1GT/1HW indicates 1 month after regular GT or HW ingestion; 2GT/2HW indicates 2 month after regular GT or HW ingestion; 3GT/3HW indicates 3 month after regular GT or HW ingestion; 4GT/4HW indicates 4 month after regular GT or HW ingestion. Data are presented as means ± standard deviations from the means (SD). One‐way analysis of variance (one‐way ANOVA) followed by Tukey's test were used to analyze changes in the measured parameters; ^aaa^ *P* < 0.001, ^aa^ *P* < 0.01 and ^a^ *P* < 0.05 (comparing values by the end of each month of the 8‐month study period with the baseline value); ^bbb^ *P* < 0.001, ^bb^ *P* < 0.01, and ^b^ *P* < 0.05 (comparing values after the 1st, 2nd, 3rd, and 4th months of the second period of intervention with the level by the end of the 4th month of the first period of intervention).

The effects of GT treatment over 4 months on SBP, DBP, and PP in the two study groups are summarized in Figure 1A and B. In Grp1, GT treatment significantly lowered SBP from baseline values by 3.3 (*P* < 0.05), 5.4 (*P* < 0.01), 8.4 (*P* < 0.001), and 10.7 mm Hg (*P* < 0.001) after the 1st, 2nd, 3rd, and 4th months of treatment, respectively, therefore resulting in a 6.6% reduction by the end of the 4th month of regular ingestion of GT. Stopping GT treatment and drinking just HW elevated SBP significantly (*P* < 0.001) by 7.5 mm Hg (4.9%) over 4 months from this final value to a level indistinguishable from the baseline (*P* > 0.05). DBP correspondingly fell insignificantly by 1.9 (*P* > 0.05), and 3.2 (*P* > 0.05), and significantly by 3.9 (*P* < 0.05), and 5.2 (*P* < 0.01) to give an overall 5.1% fall. Similarly, cessation of GT treatment and drinking only HW significantly raised (*P* < 0.05) the DBP by 3.5 mm Hg (3.6%) from the value 4 months after GT treatment to a level statistically indistinguishable from baseline values (*P* > 0.05). Finally, PP fell insignificantly by 1.4 (*P* > 0.05), 2.2 (*P* > 0.05), and 4.4 (P > 0.05), and significantly by 5.5 (*P* < 0.05), respectively, to give a 9.1% reduction after 4 months, and rose again significantly (*P* < 0.05) by 4.0 mm Hg (7.3%) from the level by the end of the 4^th^ month of GT treatment to a value indistinguishable from its baseline value by the end of the 4 months of HW ingestion period (*P* > 0.05).

In Grp2, HW ingestion for 4 months resulted in variable but insignificant changes in SBP, DBP, and PP (*P* > 0.05). While SBP was insignificantly elevated from baseline values by 1.9 (*P* > 0.05), and 2.5 (*P* > 0.05) after the 1st and 3rd months of HW ingestion, it was insignificantly lowered by 2.0 (*P* > 0.05), and 2.4 mm Hg (*P* > 0.05) by the end of the 2nd and 4th months, respectively; thus resulting in an insignificant (1.5%) reduction by the end of 4 months of HW ingestion. Conversely, stopping HW ingestion and treatment with GT for 4 months significantly (*P* < 0.001) reduced SBP by 8.8 mm Hg (5.4%) and 6.4 mmHg (4.0%) from baseline levels and values 4 months after HW ingestion, respectively. DBP was decreased insignificantly by 2.7 (*P* > 0.05), 3.9 (*P* > 0.05), and 1.0 (*P* > 0.05) following the 1st, 2nd, and 4th months, respectively; thus, giving an insignificant (1.0%) reduction by the end of month 4 of HW ingestion. On the other hand, DBP was insignificantly elevated by 1.9 mmHg (*P* > 0.05) by the end of the 3^rd^ month of HW ingestion. It was, however, significantly lowered, upon GT treatment for 4 months, by 4.2 mmHg (4.1%; *P* < 0.05), and 3.1 mmHg (3.1%; *P* < 0.05) from baseline levels and values 4 months after HW ingestion, respectively. HW ingestion resulted in insignificant elevations of PP by 4.7 mmHg (*P* > 0.05), 1.9 (*P* > 0.05), and 0.6 mmHg (*P* > 0.05) by the end of the 1st, 2nd, and 3rd months, respectively. However, PP felt insignificantly by 1.4 mmHg (2.3%) by the end of the 4th month of HW ingestion. It then declined significantly (*P* < 0.05) by 4.6 mm Hg (7.7%), and 3.3 mmHg (5.5%; *P* < 0.05) by the end of the 4 months of GT treatment period, from baseline values and levels 4 months after HW ingestion, respectively.

**Figure 2:**
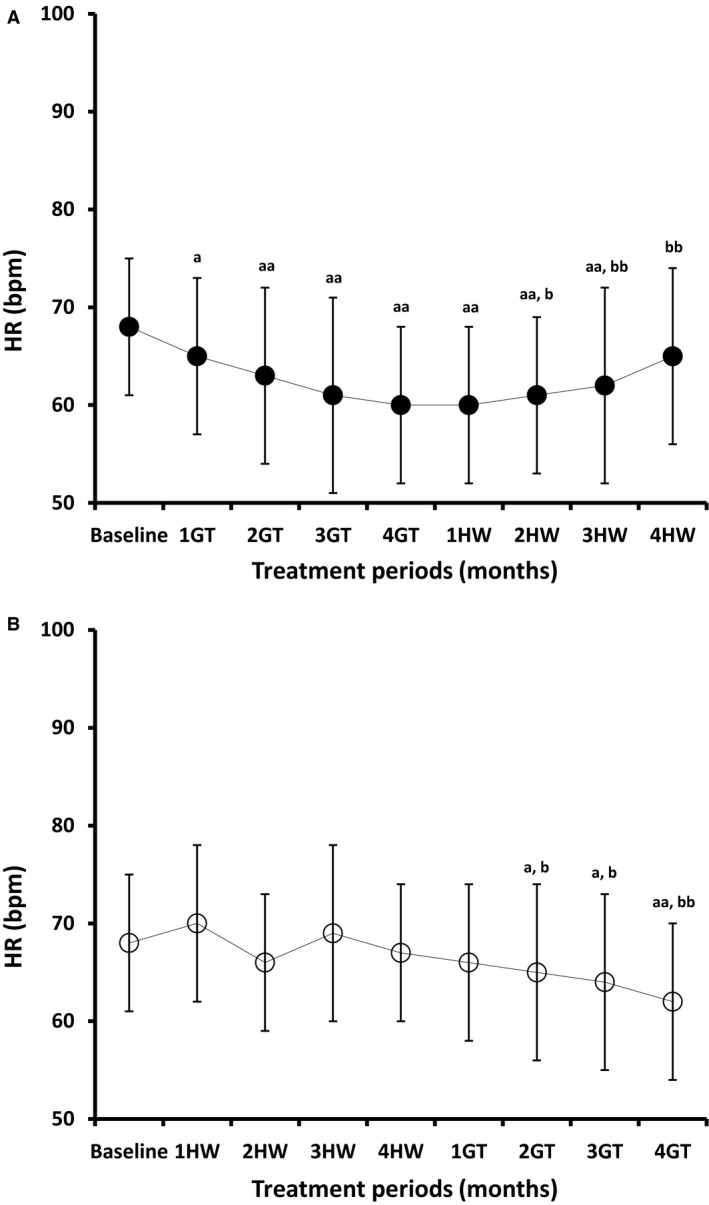
Effects of GT treatment and HW ingestion on HR (beat/min; bpm). Changes in Heart rate for Grp1*(2A)* and Grp2 *(2B)* following two periods of GT and HW intervention for 4 months each; Grp1 had GT treatment followed by HW ingestion and Grp2 had HW ingestion followed by GT treatment. During each intervention period, patient were instructed to drink either 4 standard cups (250 mL each) of GT or HW per day for 4 months from left to right in sequence. 1GT/1HW indicates 1 month after regular GT or HW ingestion; 2GT/2HW indicates 2 month after regular GT or HW ingestion; 3GT/3HW indicates 3 month after regular GT or HW ingestion; 4GT/4HW indicates 4 month after regular GT or HW ingestion. Data are presented as means ± standard deviations from the means (SD). One‐way analysis of variance (one‐way ANOVA) followed by Tukey's test were used to analyze changes in the measured parameters; ^aa^ *P* < 0.01 and ^a^ *P* < 0.05 (comparing values by the end of each month of the 8‐month study period with the baseline value); ^bb^ *P* < 0.01 and ^b^ *P* < 0.05 (comparing values after the 1st, 2nd, 3rd, and 4th months of the second period of intervention with the level by the end of the 4th month of the first period of intervention).

Figure 2A and B demonstrate the effects of GT treatment on HR over 4 months in the two study groups. GT consumption significantly and progressively lowered the HR of Grp1 by 3 (*P* < 0.05), 5 (*P* < 0.001), 7 (*P* < 0.001), and 8 beat/min (*P* < 0.001) to give a final, 11.7% reduction. However, by the end of the 4 months’ period of HW ingestion, the HR of Grp1 was significantly (*P* < 0.001) raised by 5 beat/min (8.3%) from the value 4 months after GT treatment to reach a level statistically indistinguishable from baseline value (*P* > 0.05).

In parallel with the variable changes induced by HW ingestion, the HR of Grp2 was insignificantly raised by 2 (*P* ****>** **0.05), 1 (*P* ****>** **0.05) following the 1st and 3rd months and insignificantly lowered by 2 (*P* ****>** **0.05), and 1 beat/min (*P* ****>** **0.05) by the end of the 2nd and 4th months of HW ingestion. Upon stopping HW ingestion and initiating GT treatment HR was significantly (*P* ****<** **0.001) lowered by 6 beat/min (8.8%) from its baseline values and 5 beat/min (10.1%) from levels 4 months after HW ingestion.

Thus, regular GT treatment had similar negative chronotropic influence on the HR in the Grp1 and Grp2; HR fell by 11.7% and 8.8% (*P* ****<** **0.001) from baseline level by the end of the 4^th^ month of treatment in Grp1 and Grp2, respectively.

Table 1 demonstrates that before GT treatment and HW ingestion, 20 patients (20%) of Grp1, and 24 patients (24%) of Grp2 had ECG and echocardiographic evidence of LVH. By the end of the first 4 months intervention period, the number of patients with LVH decreased significantly (*P* ****<** **0.01; Fisher's exact test) to eight patients (8%) with GT treatment, whereas the number of patients with LVH in Grp2 remained the same following HW ingestion. The number of patient with LVH in Grp1, however, increased significantly (*P* ****<** **0.01) to 16 patients (16%) upon cessation of GT treatment and over the second 4 months of HW intervention period; thus, returning the incidence of LVH to pretreatment levels (*P* ****>** **0.05). On the other hand, the number of patients with LVH in Grp2 was significantly (*P* ****<** **0.01) lowered to 10 patients (10%) by the end of the 4th month of GT treatment.

**Table 1:** Effects of GT treatment and HW ingestion on electrocardiographic and echocardiographic left ventricular hypertrophy (LVH)

	Grp1 (n = 100)	Grp2 (n = 100)
No of cases with LVH at the beginning of the study (baseline LVH)	20 (20%)	24 (24%)
No of cases with LVH by the end of the first intervention period	8 (8%)**	24 (24%)
No of cases with LVH by the end of the second intervention period	16 (16%)++	10 (10%)++ ^,^ §§

Data are presented as number of cases having LVH at the beginning of the study (baseline LVH), and by the end of the first and second intervention periods. Comparison used the Fisher's exact test; *P < 0.05, **P < 0.01 (comparing LVH by the end of the first intervention period with baseline LVH); ^+^ *P* < 0.05, ^++^ *P* < 0.01 (comparing LVH by the end of the second intervention period with LVH by the end of the first intervention period); ^§^ *P* < 0.05, ^§§^ *P* < 0.01 (comparing LVH by the end of the second intervention period with baseline LVH).

## Discussion

Hypertension is an important modifiable risk factor underlying the high incidence of CVD complications in adults (Mancia et al. 2016; Willey et al. 2016; Qin et al. 2017). The Framingham study reported reductions in the DBP in the elderly thereby elevating the PP (Franklin et al. 1999). Such reduction in DBP after the age 60 could be attributed to the reduced compliance of large arteries. On the other hand, the decreased aortic compliance elevates the SBP, which in turn increases left ventricular afterload and results in LVH with consequent diastolic followed by systolic dysfunction and LVF (Masugata et al. 2011; Okin et al. 2017). Thus, an increase in PP may be more predictive of cardiovascular risk than either SBP or DBP alone and that ideal antihypertensive therapy should be aimed at rigorous control of both SBP and DBP in order to maintain the PP within in a normal narrow range (Protogerou et al. 2007). Furthermore, LVH increases the risk of arrhythmias and sudden cardiac death independent of the level of arterial pressure (Nepper‐Christensen et al. 2017).

Tea, particularly green tea (GT) contains substantial concentrations of polyphenolic flavonoid compounds (Hodgson et al. 1999; Bøhn et al. 2012). Several animal and clinical studies have reported reduced risk of CVD in subjects with a high flavonoid intake (Hodgson et al. 1999; Bøhn et al. 2012). Animal studies have shown that GT has a strong hypotensive effect in rats (Potenza et al. 2007; Szulińska et al. 2017). On the other hand, the few human clinical trials on the effects of GT on blood pressure have reported conflicting results (Hodgson et al. 1999; Yang et al. 2004). In one study, five cups of either GT or black tea daily for 1 week did not significantly alter blood pressure (Hodgson et al. 1999). In another study, habitual moderate strength GT consumption, 120 mL/d or more for 1 year, significantly reduces the risk of developing hypertension (Yang et al. 2004). The inconsistency between these studies may largely be due to potential confounding lifestyle and dietary factors associated with tea drinking in the respective region based on different cultures (Peters et al. 2001).

We report a randomized controlled human study characterizing the effects of regular ingestion of GT on PP and LVH in hypertensive patients for the first time, to best of our knowledge. Blinding of clinical data was maintained during the study by ensuring that blood pressure measurements were not disclosed to participants and interpreting the electrocardiograms (ECGs) by observers blinded to other clinical data. Echocardiography was also done by cardiologists who were blinded to clinical data and ECGs findings.

This study shows that regular consumption of four standard cups (250 mL each) of GT per day for 4 months resulted in significant reductions in the SBP, DBP, and PP of both Grp1 and Grp2 over 4 months of treatment without causing hypotension. Similarly, regular consumption of GT seems to have a strong negative chronotropic influence. These effects were seen whether GT was ingested from the start as in Grp1, or following a period of HW ingestion as in Grp2. Stopping GT treatment and ingestion of just HW returned all these parameters to pretreatment values over 4 months in Grp1; thus confirming the reversibility of the hypotensive and the negative chronotropic effects of GT. On the other hand, ingestion of just HW did not induce significant changes on SBP, DBP or PP during the first 4 months intervention period in Grp2.

These results are generally consistent with the findings of several animal studies reporting hypotensive effects of GT (Potenza et al. 2007; Szulińska et al. 2017), and also the findings of the few clinical studies evaluating the effects of GT treatment of hypertension (Yang et al. 2004). Our findings, however, differ from the results of Hodgson and colleagues, who reported no significant effects of GT on blood pressure in normotensive subjects (Hodgson et al. 1999). This study is more comprehensive than other studies. First, we included two groups of hypertensive patients (Grp1 and Grp2) who were randomly assigned to drink GT for 4 months followed by HW ingestion for an additional 4‐month period in Grp1, or the opposite sequence of intervention in Grp2. This protocol ensures that each of the study groups can act as a control group for the other group, and additionally, within each group, the two intervention periods can act as control for each other. Second, the current investigation was run for 8 months and is therefore a better indicator of the effects of chronic ingestion of GT on the SBP, DBP, and PP. The study done by Hodgson and colleagues was run for 1 week only (Hodgson et al. 1999). Third, this study also characterized the effects of GT treatment on the PP, HR, and LVH in hypertensive patients for the first time.

At present, the exact mechanism by which GT reduces blood pressure remains unclear. Animal studies have shown that γ‐glutamylethylamide in GT reduces blood pressure significantly in SHR rats (Szulińska et al. 2017). Recent studies have shown that tea flavonoids improve vascular function and reverse endothelial vasomotor dysfunction in patients with coronary artery disease and diabetes mellitus (Duffy et al. 2001; Bøhn et al. 2012). The vasodilatory properties of GT flavonoids may thus explain its blood pressure lowering effects. Furthermore, recent evidence indicates that GT catechins inhibit angiotensin II‐induced vascular smooth muscle cell hypertrophy, which is a critical event in the development of hypertension (Zheng et al. 2004). The hypotensive effects of GT can be also attributed to inhibition of human 11*β*‐hydroxysteroid dehydrogenase type 1 (Hintzpeter et al. 2014). Given the strong negative chronotropic effect noted in this study, the hypotensive effect of GT may well be also mediated through a direct action on the heart.

Interestingly enough, we found for the first time that regular consumption of GT induced significant regression of LVH in the hypertensive patients, possibly by improving blood pressure and thereby reducing left ventricular pressure overload. The regression of LVH with GT treatment might also be the result of a direct action of GT on the myocardium. These findings are in agreement with recent animal studies confirming that chronic administration of GT attenuates hypertension and induces early reversal of angiotensin II‐induced cardiac hypertrophy and LVH in SHR rats (Papparella et al. 2008; Jin et al. 2017), and rats subjected to remnant kidney surgery (Priyadarshi et al. 2003). However, the exact mechanism by which GT induces regression of LVH is also not yet fully understood. Interestingly, GT was shown to attenuate angiotensin II‐induced cardiac hypertrophy in rats by modulating reactive oxygen species production and the Src/epidermal growth factor receptor/Akt signaling pathway (Papparella et al. 2008). The antihypertrophic effect of GT shown in this study could thus be explained by its ability to ameliorate the hemodynamic stress on the left ventricle through the reductions it caused in the afterload. It was very interesting to see regression of LVH within a relatively short period of time and specifically by the fourth month of regular GT ingestion. This agrees with a recent study in a rabbit model of LVH, where aortic banding for 8 weeks resulted in robust LVH as a result of pressure overload. Such LVH was subsequently reversed completely or partially within 8 weeks following debanding of the ascending aorta (Zhao et al. 2013). It was recently reported that treatment with the cardioselective *β*‐blocker esmolol induces some degree of regression of LVH within 48 h in an experimental rat model of primary hypertension (Quintana‐Villamandos et al. 2013).

## Conclusion

This study suggests that regular ingestion of GT has cardiovascular protective effects. However, further studies are required to review the mechanisms of the antihypertensive effects and elucidate the mechanisms of LVH regression effects of GT.

### Potential limitations of the study

Allowing the hypertensive patients to continue their antihypertensive therapy during the study could have resulted into cointervention bias. However, designing the study in such a way to include periods of both GT treatment and HW ingestion and subsequently comparing the results at least partially eliminated that bias since antihypertensive therapy continued during both periods. Differences in adherence to the planned GT treatment regimen and HW ingestion across the two study groups could have resulted into compliance bias with potential effects on the results. This was minimized by maintaining rigorous regular contact with the participants throughout the two intervention periods. Furthermore, the study design and requirements were thoroughly explained to the participants at the beginning of the study. Moreover, the participants were seen at the end of each month of the first 4 months intervention period as describe above and the importance of compliance to the GT treatment and HW ingestion protocol was stressed upon and emphasized in each visit.

## Conflict of Interest

The authors report no conflicts of interest. The author alone is responsible for the content and writing of the article.
